# Differential regulation of antioxidant enzymes in *Frankliniella occidentalis* (Thysanoptera: Thripidae) exposed to thermal stress

**DOI:** 10.7717/peerj.12089

**Published:** 2021-08-27

**Authors:** Jia-Wen Yuan, Yutao Zheng, Ya-Wen Chang, Jing Bai, Jing Qin, Yu-Zhou Du

**Affiliations:** 1 College of Horticulture and Plant Protection & Institute of Applied Entomology, Yangzhou University, Yangzhou, China; 2 Joint International Research Laboratory of Agriculture and Agri-Product Safety, Yangzhou University, Yangzhou, China

**Keywords:** *Frankliniella occidentalis*, Thermal stress, Oxidative defense, Enzymatic activity, Gene expression

## Abstract

*Frankliniella occidentalis* is an invasive insect pest that incites damage to ornamental and agronomic crops on a global scale. In this study, the effects of temperature on gene expression and enzyme activity were studied for superoxide dismutase (SOD), peroxidase (POD), and glutathione-S-transferase (GST) in *F. occidentalis*. SOD, POD and GST enzyme activity increased significantly at 35–37 °C but declined as the temperature increased to 41 °C. In a time course study at 35 °C, SOD, POD and GST activities were significantly elevated at 0.5, 1 and 2 h in comparison to the control at 26 °C. Expression patterns were evaluated for the three antioxidant genes under high and low temperature stress. In a time course study at –4 °C, *SOD*, *POD* and *GST* expression peaked at 1 h and declined at 2 h of exposure. In contrast, when transcription was monitored at 35 °C, expression was lowest at 1 h and increased at 2 h. The results provide data that will be useful in deciphering the role of antioxidant enzymes in the adaptation of *F. occidentalis* to climate change.

## Introduction

Temperature impacts the reproduction, development, and distribution of insects (Cossins & Bowler, 1987; Worner, 1998; Bale et al., 2002), and extreme temperatures are known elicitors of reactive oxygen species (ROS) in invertebrates. The excessive generation of ROS can damage cellular constituents, including lipids, proteins, and nucleic acids (Halliwell, 1989; Kamata & Hirata, 1999; Foyer & Noctor, 2005; Lopez-Martinez et al., 2008). In order to survive, insects reduce or detoxify ROS through the action of antioxidants; these function as enzymatic and non-enzymatic scavengers that reduce lipid peroxidation and decrease damage to nucleic acids and proteins (Felton & Summers, 1995; Lyakhovich et al., 2006; Krishnan et al., 2007). Peroxidase (POD), superoxide dismutase (SOD), and glutathione-S-transferase (GST) are antioxidant enzymes that defend cells from excessive levels of ROS (Felton & Summers, 1995; Wang, Oberley & Murhammer, 2001; Dubovskiy et al., 2008; Liu et al., 2020). SOD functions by degrading superoxide anions to hydrogen peroxide (H_2_O_2_) and oxygen, and H_2_O_2_ is subsequently converted to H_2_O by POD (Kashiwagi et al., 1997; Wang & Li, 2002; Liu & Ma, 2007). GSTs function to detoxify compounds that are produced from lipid peroxidation (Ahmad et al., 1991; Kono & Shishido, 1992; Dubovskiy et al., 2008).

The western flower thrips (WFT), *Frankliniella occidentalis*, damages both vegetables and ornamental plants on a global scale and is especially problematic in greenhouses (Morse & Hoddle, 2006; Kirk & Terry, 2015; Mouden et al. 2017). In addition to direct damage, WFT causes serious damage to plants by transmitting plant viruses such as the Tomato Spotted Wilt Virus (Pappu, Jones & Jain, 2009; Tomitaka, 2019). WFT is endemic to the western region of North America and has spread globally due to the transportation of agricultural products (Reitz, 2009; Kirk & Terry, 2015). According to the CABI Invasive Species Compendium, *F. occidentalis* has been discovered on all continents except Antarctica (https://www.cabi.org/isc/datasheet/24426). In mainland China, *F. occidentalis* was initially found in Beijing in 2003 (Zhang et al., 2003) and has since been discovered in at least ten provinces (Wu et al., 2017).

Previous studies indicated that temperature impacts development, sex ratios, reproduction, population growth, and mortality of *F. occidentalis* (Li et al., 2007; Li et al., 2011a; Zhang et al., 2012). During the hot summers in subtropical China, high temperatures may cause oxidative stress to *F. occidentalis*, particularly in greenhouses (Wang et al., 2014). Previous studies demonstrated that the expression of genes encoding catalase (CAT) and subsequent enzymatic activity were altered in *F. occidentalis* exposed to hot and cold stress (Shi et al., 2013; Qin et al., 2017). However, the impact of high and low temperatures on other antioxidant enzymes in *F. occidentalis* is unclear.

In this study, we investigated the effect of temperature stress on POD, SOD, and GST in *F. occidentalis*. The results provide important data on how antioxidant enzymes counteract oxidative damage in the WFT and provide a more comprehensive framework for understanding thermal tolerance in *F. occidentalis*.

## Materials & Methods

### Insects and temperature treatments

*Frankliniella occidentalis* populations were collected in Hangzhou, China, in 2008 and were reared with kidney bean, *Phaseolus vulgaris Linn*, at 25 ± 0.5 °C and 70 ± 5% relative humidity with a 16:8 h light:dark photoperiod as outlined by Li et al. (2011b). Newly emerged 2^nd^ instar larvae were collected, and pools of 100 were exposed to high (31, 33, 35, 37, 39 or 41 °C) or low (0, –2, –4, –6, –8 and –10 °C) temperatures for 1 h in glass tubes as described (Chang et al., 2017). Through the results of the pre-experiment, 35 and –4 °C were decided as the model temperature on *F. occidentalis*, which was further explored by subjecting groups of individuals to 0, 0.5, 1, and 2 h of thermal stress; controls were maintained at 26 °C (0 h time point). Following thermal stress, larvae were incubated at 26 °C for 30 min and used a brush to touch it gently, thrips would be identified as surviving if it respond to the stimulus. Survivors were frozen in liquid nitrogen and stored at –80 °C for future use. Four replicate pools were used for each temperature and time period.

### Determination of enzyme activity

The assay kit used for protein extraction was from Nanjing Jiancheng Bioengineering Institute, Jiangsu, China. Treated samples were homogenized in 0.9% saline and then centrifuged at 2,500 × rpm for 10 min (Jia et al., 2011). Supernatants containing the enzyme fractions were collected, and protein content was determined using the Bradford (1976) method.

POD and SOD activities were assessed with commercially available kits (Qin et al., 2017). Absorbance values were obtained using the BioTek PowerWave HT Microplate Spectrophotometer (Bio-Tek Instruments Inc., Winooski, Vt., USA). GST activity was measured as a function of reduced glutathione (GSH) using 10 mg of cytosolic protein and 1-chloro-2,4-dinitrobenzene (CDNB; Shanghai Chem, Shanghai, China) as a substrate (Habig, Pabst & Jakoby, 1974; Attig et al., 2014). GST activity was determined at *A*_340_ with a microplate spectrophotometer (Shanghai Xinmao Instrument, Shanghai, China), and results are shown as μmol GSH-CDNB/min/mg protein.

### RNA isolation, partial cloning of *SOD*, *POD* and *GST1*, and qRT-PCR

The SV Total RNA Isolation System was used to isolate RNA from *F. occidentalis* as recommended by the manufacturer (Promega, San Luis Obispo, CA, USA). RNA quality and concentration were determined, and cDNA was generated from total RNA with the First Strand cDNA Synthesis Kit (Clontech, Mountain View, CA, USA) as outlined previously (Zhang et al., 2019).

Transcriptome sequencing was performed on *F. occidentalis* exposed to low temperature (–13 °C), high temperature (40 °C) and normal temperature control (26 °C) at the Shanghai Biotechnology corporation by Illumina sequencing platform. The RNA-seq data were deposited with the Sequence Read Archives PRJNA73493 at NCBI. To remove adapter contamination, low-quality bases and bases artificially introduced during library construction, we trimmed all raw reads using Trimmomatic 0.32 (http://www.usadellab.org/cms/index.php?page=trimmomatic) before transcript assembly, while the unpaired reads were discarded (Bolger, Lohse & Usadel, 2014). The clean reads were mapped to the sequences in the rRNA database of all published insects downloaded from NCBI to discard rRNAs using SOAP. Only the clean reads with the standard of Q30 > 85% and processed with Trimmomatic 0.32 and SOAP were used for further analysis (Gu et al., 2019). Clean data were assembled with Trinity to obtain a high-quality unigene library (Grabherr et al., 2011). The unigene library of three species were first assembled to obtain individual UniGene databases; the general UniGene library was obtained by clustering the three individual databases through CD-Hit to facilitate comparison of expression patterns (Fu et al., 2012). The transcripts selected in the clustering united as unigenes using the De Bruijn graph algorithm; CD-Hit was used to reduce sequence redundancy and improve the performance of other sequence analyses (Fu et al., 2012; Yang & Smith, 2013). For functional annotation, we obtained information on unigenes using BLAST (cut-off e-value of 10–5) with protein databases such as NR (NCBI nonredundant database), Swiss-Prot, GO (Gene Ontology), COG (Clusters of Orthologous Groups), KOG (euKaryotic Orthologous Groups), eggNOG, Pfam (Protein family) and KEGG (Kyoto Encyclopedia of Genes and Genomes). “Superoxide Dismutase”, “Peroxidase” and “glutathione-s-transferase” were used as key words to search related gene fragments in the transcriptome database of *F. occidentalis*, respectively. The fragment gene cDNA of the *SOD*, *GST1* and *POD* was submitted to GenBank (accession no. MZ364120, MZ364118 and MZ364119, respectively). According to the obtained gene fragments, the corresponding primers (Table 1) were designed for fragment verification. PCR products were cloned and sequenced as described (Zhang et al., 2019).

**Table 1:** Primers used in the study.

Primer name	Primer sequences	Tm (°C)	Length (bp)	E^c^ (%)
DP-*SOD*-F	AATGCTGCGTTCTCTGTTGTG	58.7	335	
DP-*SOD*-R	TCTGGTTTTGTTGTTTCAGGAGT	58.4		
DP-*POD*-F	CAACCCCGACCAGCCCTAC	62.3	600	
DP-*POD*-R	AAAAGGGGAAATCGGTGTCG	61.4		
DP-*GST*-F	TGACCGTGAACCAGACCGAG	61.3	431	
DP-*GST*-R	GATGCCGAAAATACTGAGTGTGG	61.4		
qPCR-*SOD*-F	GAAATAACTGGTTCCAAGGCACT	59.6	125	91.8
qPCR-*SOD*-R	AATGCTGCGTTCTCTGTTGTG	58.7		
qPCR-*POD*-F	CCGCACTGGGACGACGAGAC	65.8	235	96.4
qPCR-*POD*-R	CGATGAGCGAGTGGAAGTATCTGAA	64.8		
qPCR-*GST*-F	GCTGCTGCTGTGCTGGATTA	59.7	170	90.0
qPCR-*GST*-R	ACCGTGAACCAGACCGAGAC	59.4		
*EF-1*-F	TCAAGGAACTGCGTCGTGGAT	58.6	130	95.4
*EF-1*-R	ACAGGGGTGTAGCCGTTAGAG			
*18S*-F	AACACGGGAAACCTCACCA	55.4	116	108.9
*18S*-R	CAGACAAATCGCTCCACCAA			
*RPL32*-F	CAACATCGGTTATGGAAGCA	55.0	141	100.1
*RPL32*-R	ACAGCGTGGGCAATTTCAGC			
*GAPDH*-F	AAGGGTGCTCAGGTTGTTGCT	56.5	89	104.4
*GAPDH*-R	CGACCGTGGGTGGAGTCATAT			

Quantitative real-time reverse transcriptase PCR (qRT-PCR) was conducted using the protocols described by Zhang et al. (2019). Specific primers (Table 1) were designed according to the above verified fragments for qRT-PCR. Melting curve analysis was executed to analyze the specificity of PCR products. According to the evaluation results of Zheng et al. (2014) on the reliability of reference genes in *F. occidentalis*, expression levels were normalized using reference genes *GAPDH, RPL32* and *EF-1, 18S* for high and low temperature stress, respectively.

### Statistical analyses

qRT-PCR data analyzing was conducted in Bio-Rad CFX Manager 3.1 software. The average Ct values of biological replicates were used to calculate the relative expression levels. The results of qPCR were analyzed with the 2^–ΔΔCt^ method (Livak & Schmittgen, 2001). Firstly, for all test samples and calibration samples, the Ct value of the housekeeping gene were used to normalize the Ct value of the target gene. Normalized results were ΔCt(test) and ΔCt (calibrator), respectively. And using ΔCt (calibrator) to normalize ΔCt (test), ΔΔCt was obtained. The ratio of expression level was calculated by 2^–ΔΔCt^. The lg(X) method was used to transform the expression level data for normality and homogeneity of variance. Significant differences were detected by one-way analysis of variance (ANOVA) and Duncan’s multiple comparisons test. Data were analyzed with SPSS v. 16.0 and considered significant at *P* < 0.05.

## Results

### Effect of high temperature stress on antioxidant activity

**Figure 1:**
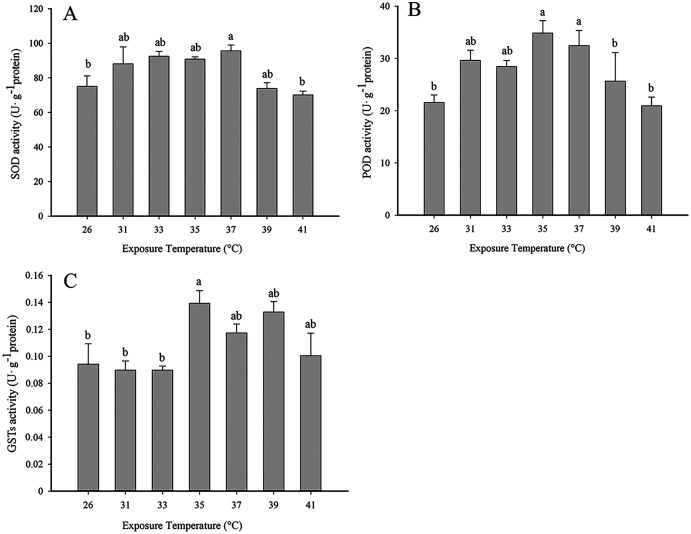
Effect of high temperature stress on antioxidant enzyme activity in 2^nd^ instar larvae of *F. occidentalis*. (A) SOD, superoxide dismutase; (B) POD, peroxidase; (C) GST, glutathione-S-transferase. Larvae were exposed to 31, 33, 35, 37, 39, and 41 °C for 1 h in glass tubes; 26 °C was used as the control. Each value represents the mean (±SE) of four replications. Columns labeled with different letters indicate significance at *P* < 0.05 using ANOVA (Ducan’s test).

SOD activity increased with rising temperature from 31 to 37 °C and was highest at 37 °C. The activity of SOD activity began to decline at 39 °C, and the level at 41 °C was significantly lower than 37 °C (*F*_6,19_ = 4.245, *P* < 0.05) (Fig. 1A). A similar pattern was observed with POD, where activity rose with increasing temperature, peaked at 35 °C and was significantly lower at 41 °C than 35 °C (*F*_6,21_ = 7.089, *P* < 0.05) (Fig. 1B). GST activity was highest at 35 °C (Fig. 1C) and began to decline with increasing temperature (*F*_6,21_ = 8.312, *P* < 0.05).

### Temporal changes in antioxidant enzyme activity at 35 °C

**Figure 2:**
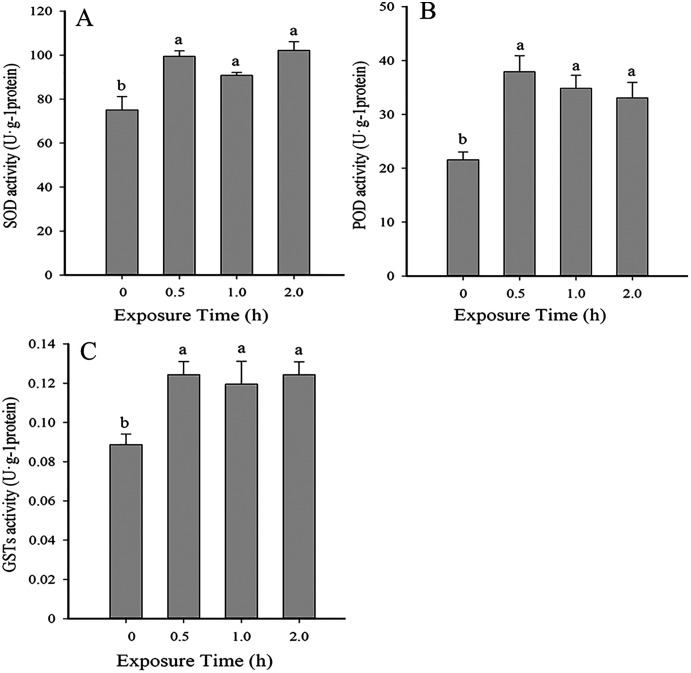
Temporal changes in antioxidant enzyme activity in 2^nd^ instar larvae of *F. occidentalis* exposed to 35 °C. (A) SOD, superoxide dismutase; (B) POD, peroxidase; (C) GST, glutathione-S-transferase. *F. occidentalis* was exposed to 35 °C for 0.5, 1, and 2 h and then analyzed for enzyme activity. The control group was maintained at 26 °C (0 h time point). Columns show the mean (±SE) of four replications, and columns labeled with different letters indicate significance at *P* < 0.05 in ANOVA (Ducan’s test).

Antioxidant enzyme activity was significantly higher than the control (0 h, 26 °C) when insects were exposed to 35 °C for 0.5, 1 and 2 (SOD: *F*_3,10_ = 10.005, *P* < 0.05; POD*: F*_3,12_ = 8.037, *P* < 0.05; GSTs*: F*_3,10_ = 5.815, *P* < 0.05). No significant differences in antioxidant activity were detected between 0.5, 1 and 2 h of exposure (Fig. 2).

### Expression of antioxidant genes in response to heat and cold stress

**Figure 3:**
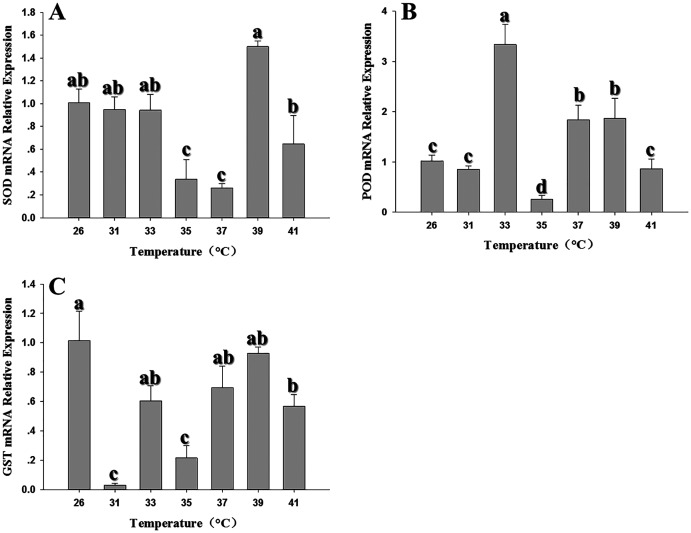
Effect of high temperature stress on expression of antioxidant genes in 2^nd^ instar larvae of *F. occidentalis*. (A) SOD, superoxide dismutase; (B) POD, peroxidase; (C) GST, glutathione-S-transferase. Larvae were exposed to 31, 33, 35, 37, 39, and 41 °C for 1 h in glass tubes; 26 °C was used as the control. Expression levels were normalized with respect to *GAPDH*. Values represent the mean (±SE) of four replications, and columns labeled with different letters indicate significance at *P* < 0.05 in ANOVA (Ducan’s test).

The expression of antioxidant genes was evaluated at 31, 33, 35, 37, 39 and 41 °C; 26 °C served as a control. *SOD* expression showed significant decreases in expression at 35–37 °C; however, expression peaked at 39 °C and was comparable to the control (26 °C) (Fig. 3A). With the exception of 35–37 °C, *SOD* expression was not significantly changed by high temperatures (*F*_6,18_ = 29.203, *P* < 0.05). In contrast, *POD* expression levels at 33, 37 and 39 °C were significantly higher than the control at 26 °C; however, except that the expression level was significantly decreased at 35 °C, there was no significant difference in the expression level of 31 °C compared with the control (*F*_6,18_ = 51.745, *P* < 0.05) (Fig. 3B). *GST1* expression was suppressed or unaffected relative to the control at all elevated temperatures (*F*_6,17_ = 32.682, *P* < 0.05) (Fig. 3C). All three antioxidant genes shared a common insensitivity in response to high temperature. Even under some temperatures, the expression level was higher than that of the control, but the relative expression level was not very high.

**Figure 4:**
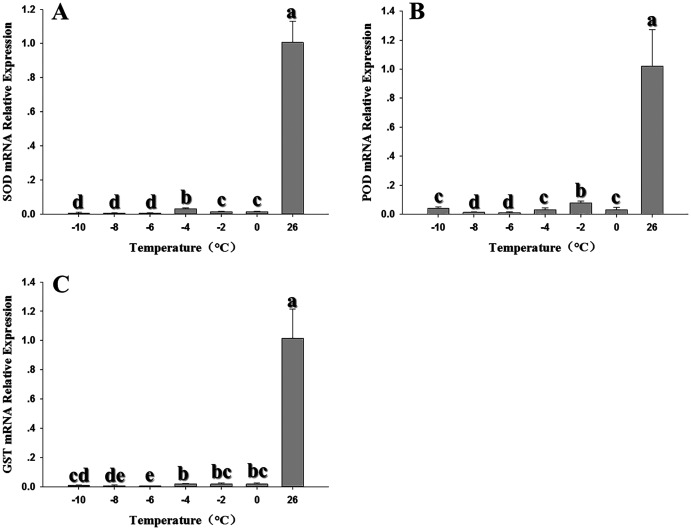
Effect of low temperature stress on expression of antioxidant genes in 2^nd^ instar larvae of *F. occidentalis*. (A) SOD, superoxide dismutase; (B) POD, peroxidase; (C) GST, glutathione-S-transferase. Larvae were exposed to 0, −2, −4, −6, −8 and −10 °C for 1 h in glass tubes; 26 °C was used as the control. Expression levels were normalized with respect to *EF-1*. Values represents the mean (±SE) of four replications, and columns labeled with different letters indicate significance at *P* < 0.05 in ANOVA (Ducan’s test).

Expression of the three antioxidant genes was also evaluated in response to low temperature stress at 0, –2, –4, –6, –8 and –10 °C. *SOD* expression showed a significant decline at all temperatures relative to the control at 26 °C; although the expression of –4 °C was higher than other temperatures, it was also inhibited by low temperature (Fig. 4A) (*F*_6,20_ = 243.607, *P* < 0.05). *POD* expression was also strongly inhibited, with the lowest expression appeared at –6 °C. (*F*_6,18_ = 51.909, *P* < 0.05) (Fig. 4B). Like *SOD* and *POD*, the *GST1* expression were decreased compared with the control relative with low temperature. (*F*_6,17_ = 32.682, *P* < 0.05) (Fig. 4C).

### Temporal changes in the expression of antioxidant genes

**Figure 5:**
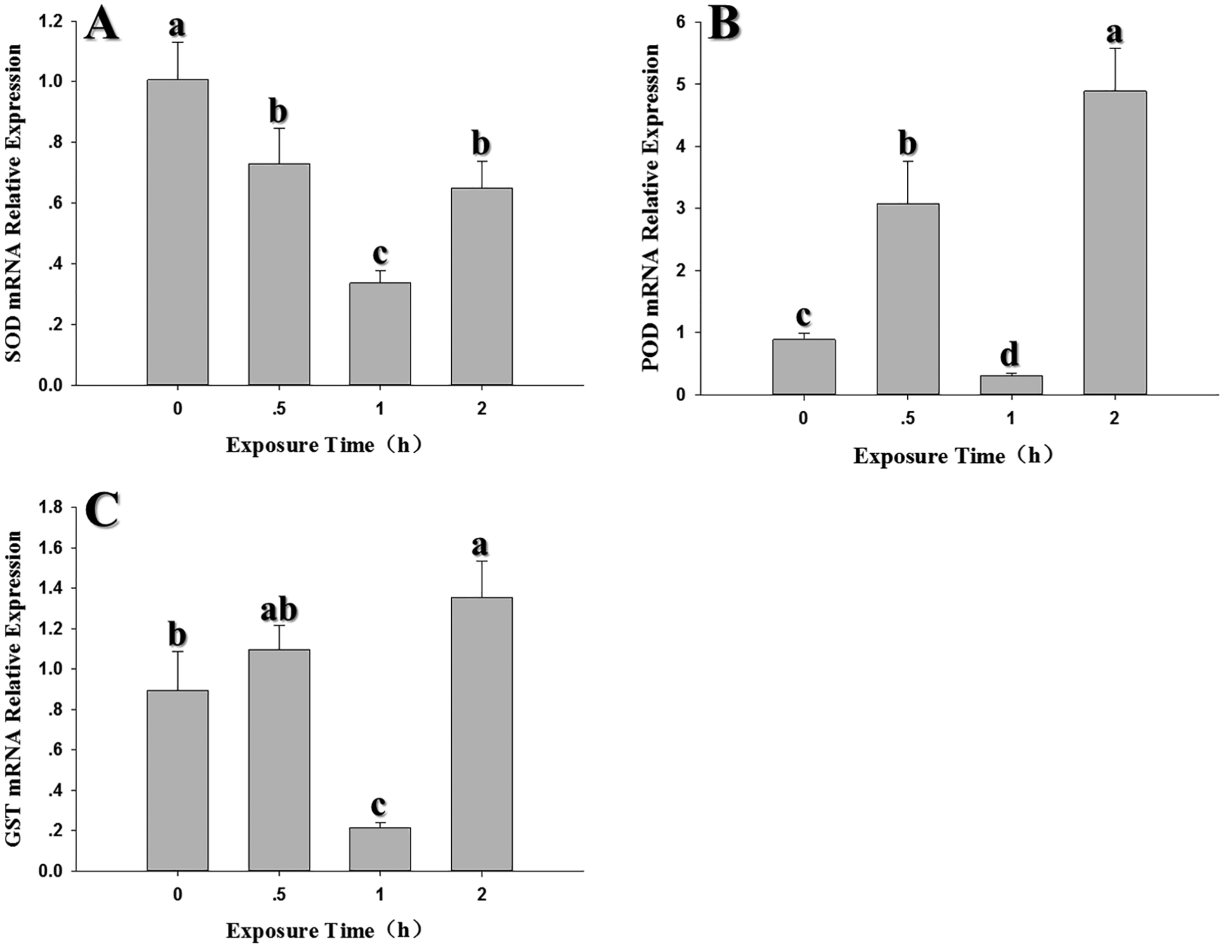
Temporal changes in the expression of antioxidant genes in 2^nd^ instar larvae of *F. occidentalis* exposed to 35 °C. (A) SOD, superoxide dismutase; (B) POD, peroxidase; (C) GST, glutathione-S-transferase. *F. occidentalis* was exposed to 35 °C for 0.5, 1, and 2 h and then analyzed for gene expression; the control group was maintained at 26 °C (0 h time point). Expression levels were normalized with respect to *GAPDH*. Columns show the mean (±SE) of four replications, and columns labeled with different letters indicate significance at *P* < 0.05 in ANOVA (Ducan’s test).

Compared to the control (0 h, 26 °C), *SOD* expression decreased significantly when 2^nd^ instar larvae were exposed to 35 °C for 0.5, 1 and 2 h (*SOD: F*_3,12_ = 31.689, *P* < 0.05) and was lowest at the 1 h exposure period (Fig. 5A). *POD* expression was significantly upregulated at 0.5 and 2 h and was higher than expression levels at 0 (control) and 1 h, with the peak appeared at 2 h. (*F*_3,12_ = 72.243, *P* < 0.05) (Fig. 5B). *GST1* expression pattern was similar to POD (Fig. 5C). Although there was no significant difference at 0.5 h compared to the control (*GST1: F*_3,11_ = 1709.476, *P* < 0.05).

**Figure 6:**
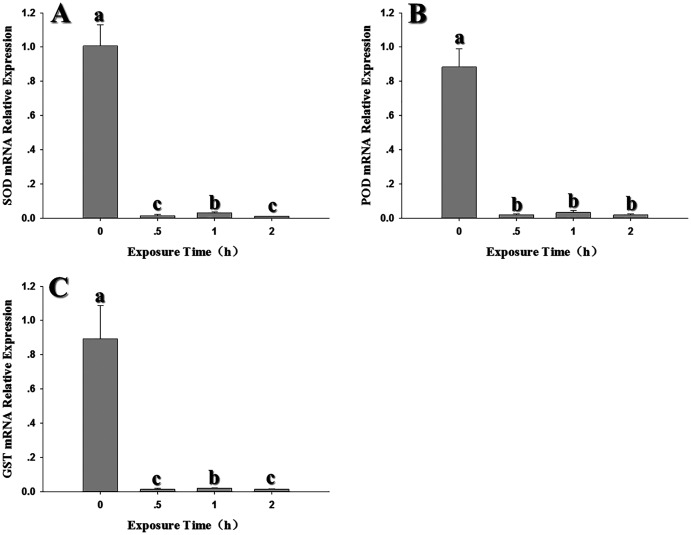
Temporal changes in the expression of antioxidant genes in 2^nd^ instar larvae of *F. occidentalis* exposed to −4 °C. (A) SOD, superoxide dismutase; (B) POD, peroxidase; (C) GST, glutathione-S-transferase. *F. occidentalis* was exposed to 4 °C for 0.5, 1, and 2 h and then analyzed for gene expression; the control group was maintained at 26 °C (0 h time point). Expression levels were normalized with respect to *EF-1*. Columns show the mean (±SE) of four replications, and columns labeled with different letters indicate significance at *P* < 0.05 in ANOVA (Ducan’s test).

After exposure to −4 °C, the expression levels of the three antioxidant genes decreased significantly when compared to the control (*SOD: F*_3,10_ = 201.898, *P* < 0.05; *POD: F*_3,11_ = 204.420, *P* < 0.05; *GST1: F*_3,10_ = 72.835, *P* < 0.05). Interestingly, all three genes showed a peak in expression after a 1 h exposure to −4 °C; however, it should be noted that expression at 1 h was lower than the control (Fig. 6).

## Discussion

Insects are poikilotherms that are greatly impacted by temperature fluctuations (Cossins & Bowler, 1987; Worner, 1998; Bale et al., 2002). When exposed to thermal stress, insects sustain oxidative damage at the cellular level and respond with surplus levels of ROS (Lopez-Martinez et al., 2008; Cui et al., 2011; Li & Sattar, 2019). ROS can cause direct damage to biological macromolecules and can also incite genetic mutations and cell death (Ryter et al., 2007). Antioxidant enzymes function to eliminate or reduce ROS levels in insects. Previous studies showed that SOD, POD and GST play important roles in the response of insects to ROS (Abele et al., 1998; An & Choi, 2010; Celino et al., 2011; Liu et al., 2020). In this study, SOD, POD and GST activity increased significantly in response to high temperatures, which suggests that these enzymes function to remove excess ROS during thermal stress. Thus, our results are consistent with those reported for *Bactrocera dorsalis*, *Bombyx mori*, *Mononychellus mcgregori*, *Diaphorina citri* and *Neoseiulus cucumeris* (Lee et al., 2005; Jia et al., 2011; Marutani-Hert, Hunter & Hall, 2010; Lu et al., 2014; Zhang et al., 2014). In a previous report, low temperature stress significantly altered SOD, POD, CAT and GST activity in *F. occidentalis* (Shi et al., 2013). The increase in POD activity was likely the result of elevated levels of SOD activity in response to H_2_O_2_. Although increased levels of antioxidant enzymes suggests a defensive function of these enzymes in counteracting the negative effect of ROS, there were no significant differences in SOD, POD or GST activity at 0.5, 1.0 and 2.0 h of exposure to 35 °C (Fig. 2). This might indicate that antioxidant enzyme activity is very sensitive to high temperature stress and reached a threshold level at 0.5 h or earlier.

Many researchers have shown that temperature stress can lead to changes in antioxidant gene expression in insects (Yang et al., 2019; Xia et al., 2019; Lu et al. 2017). Previous results showed that temperature stress inhibited the transcription of *SOD*, *POD, GST1* and related enzymes in *Mythimna separate*, *Apis cerana cerana* and *Helicoverpa armigera* (Shen et al., 2016; Yang et al., 2019; Xia et al., 2019). These results reflect the diversity of molecular responses in organisms exposed to external stress. In addition to recruiting antioxidant enzymes to remove ROS in response to thermal stress, insects also respond by synthesizing osmoprotectants, altering membrane lipid content, and expressing heat shock proteins (Chen & Kang, 2005). A previous study demonstrated that both high and low thermal stress induced *CAT* expression in *F. occidentalis* (Qin et al., 2017); therefore, the down-regulation of *POD* in this study might be attributed to increased expression of *CAT*. In the case of *SOD* and *GST1*, thermal stress may induce the synthesis of unknown substances that could inhibit transcription. Further research is needed to validate or disprove these conjectures.

Differential regulation of antioxidant genes and enzymes has been reported in insects; for example, *POD*, *CAT* and *SOD* expression patterns were not necessarily correlated with enzyme activity during high temperature stress in *Mononychellus mcgregori* (Lu et al. 2017). In larvae of *Bombyx mori*, carboxylesterase activity was not correlated with gene expression (Liu et al., 2010). Elevated protein levels can be stressful for the organism, and the organism may inhibit gene transcription to maintain homeostasis. Conversely, if protein levels fall to a suboptimal level, the cell may respond by promoting transcription. Furthermore, transcription is often followed by post-transcriptional processing, degradation of transcription products, translation, post-translational processing and further modifications that impact protein levels. Further research is needed to understand the mechanisms that control the response of *F. occidentalis* to thermal stress.

## Conclusions

This study reveals differential regulation of antioxidant gene expression and enzyme production in response to thermal stress. The results confirm the importance of antioxidant enzymes in modulating the response to thermal stress in *F. occidentalis*, and provide new avenues for further study of antioxidant mechanisms and physiological responses of *F. occidentalis*. The inconsistencies between gene expression and enzyme activity further illustrate the complexity of thermal adaptation in *F. occidentalis*. Future multidisciplinary research in genomics, transcriptomics, proteomics, and metabolomics will help explain the underlying mechanisms of thermal adaptation in *F. occidentalis*.

## Supplemental Information

10.7717/peerj.12089/supp-1 Supplemental Information 1 The real-time quantitative raw data of SOD, GST and POD at different temperatures and exposure times for data analysis and preparation of Figs. 3–6.

10.7717/peerj.12089/supp-2 Supplemental Information 2 The raw data of SOD, GST and POD activity at different temperatures and different exposure times for data analysis and preparation of Figs. 1 and 2.

10.7717/peerj.12089/supp-3 Supplemental Information 3 NR annotation.

10.7717/peerj.12089/supp-4 Supplemental Information 4 BLAST RESULTS WITH GST.

## Additional Information and Declarations

### Data Availability

The following information was supplied regarding data availability: The raw measurements are available in the Supplemental Files.
